# Zinc, copper, and selenium levels in vitiligo: a systematic review and meta-analysis

**DOI:** 10.1038/s41598-024-61982-8

**Published:** 2024-10-10

**Authors:** Khan Anam, Sampath Ananyan, Mittal Rishabh, Asati Dinesh, Kotnis Ashwin

**Affiliations:** 1 Department of Dermatology, All India Institute of Medical Sciences Bhopal, Bhopal, India; 2 Department of Biochemistry, All India Institute of Medical Sciences Bhopal, Bhopal, India; 3 All India Institute of Medical Sciences Bhopal, Bhopal, India

**Keywords:** [Mesh], Vitiligo, Copper, Selenium, Zinc, Oxidative stress, Genetic predisposition to disease, Biochemistry, Diseases, Medical research

## Abstract

Vitiligo is a dermatological disease characterized by loss of melanocytes, causing non-scaly white macules on the skin. Zinc, copper, and selenium are important micronutrients that play a role in the normal functioning of the body and have been found to potentially aid in vitiligo treatment, although the relationship between their serum levels and vitiligo is not yet fully understood. This is a systematic review aimed at assessing the levels of serum zinc, copper, and selenium and their association with vitiligo. This review was performed following the Preferred Reporting Items of the systematic Review and Meta-Analysis (PRISMA) checklist and Cochrane guidelines. A comprehensive literature search was conducted on PubMed, Google Scholar and 41 studies published between 1970 and 2022 including 3353 vitiligo cases and 10,638 controls were included in the meta-analysis conducted from August 2022 till September 2023. The quality of the studies was assessed using the National Heart Lung and Blood Institute Study Quality Assessment tool, and the risk of bias was represented using the RobVis tool. The statistical analysis was performed using Review Manager (RevMan) Version 5.4. This meta-analysis indicate a significant decline in serum zinc levels (Z = 4.97; *P* < 0.0001; SMD = − 0.86; 95% CI − 1.19 to − 0.52) in vitiligo group with high statistical heterogeneity (Tau2 = 0.74; Chi2 = 513.95, d.f. = 26 [*P* < 0.00001]; I2 = 95%). Similarly for serum copper levels there was decline (Z = 2.43; *P* < 0.0001; SMD = − 0.50; 95% confidence interval [CI] − 0.91 to − 0.10) in vitiligo group and high statistical heterogeneity (Tau2 = 0.92; Chi2 = 475.10, d.f. = 22 [*P* < 0.00001]; I2 = 95%). On the other hand, there was a increase of serum selenium levels in the vitiligo group (Z = 0.56; *P* < 0.0001; SMD = 0.23; 95% confidence interval [CI], 0.58 to 1.04) and the results reveals high statistical heterogeneity among studies (Tau2 = 1.93; Chi2 = 406.44, d.f. = 11 [*P* < 0.00001]; I2 = 97%) in vitiligo patients compared to healthy controls. Publication bias was not found for the studies analysed. This study analyses the association of serum micronutrient levels and vitiligo among patients and controls from published research along with sub-group analysis specific to Asian populations using a meta-analysis. Low serum levels of Zinc and copper and high selenium levels are associated with Vitiligo.

## Introduction

Vitiligo is an acquired dermatological disease characterised by selective and progressive loss of melanocytes that lead to milky white, non-scaly macules on the skin, hair and mucous membranes^1^. Vitiligo is reported in people of all ethnic groups irrespective of their race, gender & skin types^2^. Though the condition has been known for centuries, vitiligo remains a target for stigmatization and causes quality of life impairment in a large population of patients. It affects up to 1% of the world population making it the most common depigmentation disorder. Vitiligo occursinall races but is clearly more visible in dark-skinned people because of strong contrast. Emotional stress, sunburn, a major illness or surgical procedure, any physical trauma are some of the major triggers for vitiligo^3^. More than 50% of the patients develop the disease before 20 years of age. Onset of disease at later stage is unusual and also raises concerns about other autoimmune associated disorders such as thyroid dysfunction, rheumatoid arthritis, diabetes mellitus, and alopecia areata as co-morbidity with vitiligo.

Zinc (Zn), Copper (Cu) and Selenium are crucial micronutrients found in trace quantities in the human body and have major functions in homeostatic mechanisms of the body, such as specific immunity, inflammation and oxidative stress (OS)^4,5^. Zinc acts as a co-factor for over 300 metalloenzymes and over 2000 transcription factors and is responsible for the cells, tissues and human body to function normally. For healthy adults, normal zinc levels in serum ranges from 70 to 180 µg per 100 ml with RDA of 8 mg per day for women and 11 mg per day for men. Zinc performs numerous functions in our body such as in the synthesis of melanin, in immune system and in reproductive health. It is also observed that supplementation of zinc is valuable adjuvant treatment in vitiligo patients because of its cell reinforcement and against apoptotic properties^5,6^. It is seen that use of zinc and other trace elements not only regresses the size of lesions but also increase melanogenesis^7^. During melanogenesis, copper and zinc serve as metalloenzymes which therefore catalyzes 5,6-dihydroxy indole-2 carboxylic acid (DHICA) leading to eu-melanin development by causing a rearrangement in dopachrome^8^. Also due to antioxidant property of zinc, copper and other trace elements also prevents the toxic effect of free radicals^9^. It is still not known that how levels of zinc and copper are associated with sera of vitiligo patients. Many studies have reported reduction in serum Zinc^10–15^ and Copper levels^16–21^ in patients suffering from vitiligo, while on the other hand, Zaki et al. 2020, Salem et al. 2018, and several other studies states that high serum zinc levels are associated with vitiligo risk^7,18,19,22–27^. Similarly Wang et al. 2012, Yao et al. 2011 and several similar studies showed higher levels of serum copper to be associated with vitiligo risk^23,25,28–32^. The available evidence is not uniform and contradictory conclusions have been reported.

Selenium is an important mineral for normal homeostasis in human because it is present in more than 30 seleno-proteins and can influence in the immune system. Selenium plays a vital role in function of Glutathione peroxidase enzyme which plays pivotal role in redox regulation and is implicated in vitiligo pathogenesis. Selenium deficiency predisposes to several dermatological disorders and low selenium levels are observed in severe psoriasis. Selenium supplementation has been found to be useful in vitiligo therapy. Several reports concluded that the selenium level in vitiligo were found to be slightly elevated^11,33,34^, while others reported reduced levels^20,35,36^ or no difference^37,38^Therefore, such controversial reports encouraged us to conduct a meta analysis and systematic review to summarize the studies on level of serum zinc, copper and selenium levels in vitiligo patients, so as to analyse their association with vitiligo and to understand its role in pathogenesis and treatment outcome.

## Materials and method

The meta-analysis was conducted to assess the serum levels of zinc, copper and selenium in individuals with Vitiligo and analyse it association with Vitiligo. This review has been performed in concordance with the Preferred Reporting Items of the systematic Review and Meta-Analysis (PRISMA) checklist and also each step of the review has been performed as per the Cochrane guidelines.

### Review registration

This Systematic review and Metanalysis’ protocol was analysed and registered with PROSPERO (International Prospective Register of Systematic Reviews) database vide registration number CRD42022367699after confirming that no similar study assessing the effect of three metal ions, other influencing factors and vitiligo was being performed.

### Search strategy

A computer based comprehensive literature search was conducted on PubMed, Google Scholar using the search terms “vitiligo”, “vitiligo micronutrients”, “zinc”, “copper”, “selenium”, “serum zinc levels vitiligo”, “serum copper levels vitiligo” and “serum selenium levels vitiligo” studies from 3rdAugust 2022 till 10th of September 2023 and 1501 articles were found. Further, a secondary search was performed by the expert reviewer and 82 articles were found. Additionally, a manual search from the bibliography of selected studies was used to add articles if they met the standards of the selection criteria for the study and 10 articles were found.

### Selection criteria

The selection criteria were defined based on the PICO construct, where studies that included:

i. Patients with physicians’ diagnosed vitiligo with overt symptoms including localised, generalized, or universal vitiligo and stable or active vitiligo and control groups involving healthy individuals;
ii. The study participants who were subjected to biochemical analysis of their blood to reveal amounts of specific mineral micronutrients selenium, zinc and copper.
iii. All observational studies (Cohorts, Case Controls) studies with the search terms where comparators exist were included.
iv. Published, Pre-Published, Pre-Prints available as full texts online in English language published globally were included.

The selected articles were further filtered as per the pre-decided exclusion criteria if they were:

i. Case reports, Case series, Review articles of any sort, editorial or expert comments, conference presentations, authors’ response to publications or texts, incomplete papers or papers with only abstracts available and incomplete clinical trials.
ii. Animal Studies, AI models, in-vitro studies and other non-human clinical studies
iii. Studies whose full-text cannot be found online
iv. Studies with unclear, incomplete or overlapping data and evidences that cannot be reliably extracted for analysis.

### Data search

After completion of the search, articles from PubMed, Google Scholar, the collected articles (n = 1583) were screened and irrelevant studies were excluded, and remaining studies were exported onto a Microsoft Excel Spreadsheet for further screening (n = 127). Duplicates were removed (n = 80) through the Data cleanup tool using the ‘Title-Author-Date of publication’ criteria and insufficient data articles were removed, the remaining 41 articles were selected for full-text analysis (AS and AnK). The following data were collected from each study such as first author, year of publication, country, number of cases and controls, mean values and standard deviation values. General characteristics of included studies are presented in Table 1 (by AnK) and further reviewed (by AsK and AS).

**Table 1:** General characteristics of 41 included studies in meta- analysis.

S. No.	Author	Year	Ions analysed	Country	Total participants	Vitiligo cases	Healthy control
1	Archana	2021	Copper	India	120	60	60
2	Muawia	2020	Copper	Sudan	100	50	50
3	Zaki	2020	Zinc	Egypt	100	50	50
4	Saniee	2019	Zinc	Iran	196	98	98
5	Muhammad	2019	Zinc	Sudan	100	50	50
6	Narang	2018	Zinc, Copper	India	160	100	60
7	Salem	2018	Zinc, Copper	Egypt	100	50	50
8	Mirnezami	2018	Zinc	Iran	206	103	103
9	Wacewicz	2017	Zinc, Copper, Selenium	Poland	108	50	58
10	Song	2017	Selenium	China	124	62	62
11	Mogaddam	2017	Zinc	Iran	200	100	100
12	Mirza	2016	Zinc	Bangladesh	110	60	50
13	Basha	2014	Zinc	Egypt	120	60	60
14	Wang	2012	Zinc, Copper	China	240	120	120
15	Yao	2011	Zinc, Copper, Selenium	China	140	90	50
16	Wang	2011	Zinc, Copper	China	58	28	30
17	Barikbin	2011	Selenium	Iran	105	60	45
18	Zhao	2011	Selenium	China	52	36	16
19	Wu	2010	Zinc, Copper	China	140	70	70
20	Haider	2010	Zinc	Bangladesh	60	30	30
21	Ali	2010	Zinc	Bangladesh	120	60	60
22	Ozturk	2008	Selenium	Turkey	60	30	30
23	Wang	2007	Zinc, Copper	China	88	68	20
24	Ines	2006	Selenium	Tunisia	76	36	40
25	Gu	2005	Copper	China	66	30	36
26	Kang	2002	Zinc, Copper, Selenium	China	64	30	34
27	Li	2001	Copper	China	126	96	30
28	Beazley	1999	Selenium	UK	122	61	61
29	Tu	1998	Selenium	China	66	29	37
30	Tan	1997	Zinc. Copper	China	787	37	750
31	Zhou	1996	Zinc, Copper	China	46	26	20
32	Wang	1996	Zinc, Copper	China	189	48	141
33	Wang	1993	Zinc, Copper, Selenium	China	64	30	34
34	Shi	1993	Zinc, Copper	China	270	120	150
35	Tu	1991	Zinc, Copper	China	63	27	36
36	Chen	1989	Zinc, Copper	China	90	60	30
37	Li	1988	Zinc, Copper	China	142	7	135
38	Wang	1986	Selenium	China	200	100	100
39	Teherani	1986	Selenium	Austria	13	5	8
40	Genov	1972	Copper	Bulgaria	104	84	20
41	Lal	1970	Copper	India	50	30	20

### Data extraction

#### Eligible studies

**Figure 1:**
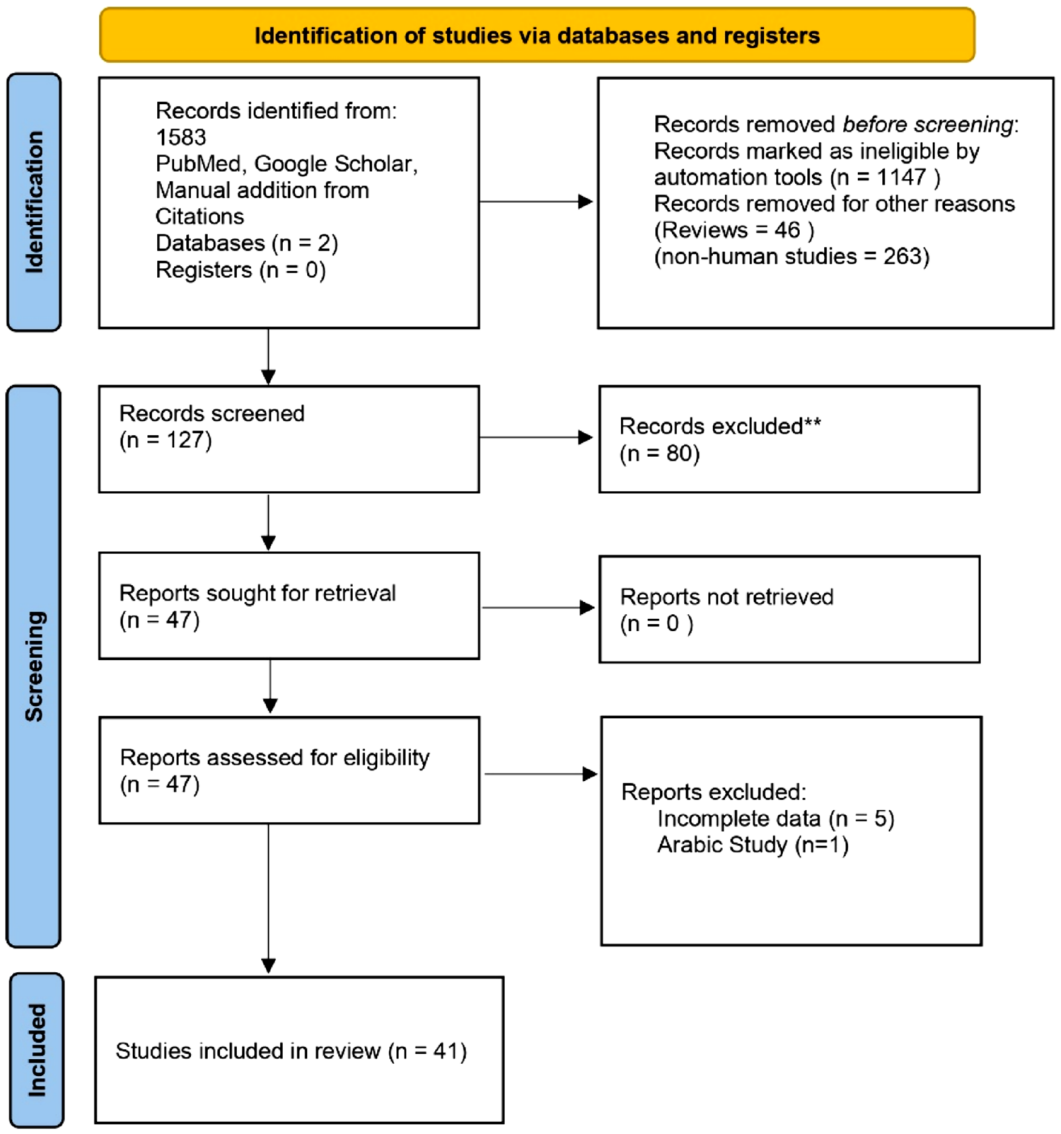
PRISMA flow diagram for included studies.

After a thorough literature search on PubMedand reference matching on Google scholar, 1583 articles were found. 1456 articles were excluded as 1147 were irrelevant to vitiligo and/or serum Zn, Cu and Se levels, 263 articles were not case- control studies and 46 were review articles. 127 articles were screened from which 80 articles were duplicate, 5 articles had insufficient data and 1 was Arabic study. 41 studies including 3353 vitiligo cases and 10,638 controls were included in the meta- analysis. The PRISMA flow diagram of the included studies is shown in Fig. 1.

### Data outcomes

The main outcome was to understand the association in change in levels of Metal Ions of copper and/or zinc and/or selenium and vitiligo. Other factors in the cascade of impact analysed were ethnicity, genetic predisposition via genetic analysis, trauma preceding vitiligo, medications and diet patterns and known oxidative stressors.

### Quality assessment

Two authors independently (AnK and AS) assessed the methodological quality of the studies included in the review by using the National Heart Lung and Blood Institute (NHLBI) Study Quality Assessment tool for case–control studies (12 questions), systematic review and meta-analysis (8 Questions); observational cohort and cross-sectional studies (14 questions)^39^.Each of the domains contained multiple questions, each of the questions could be marked as Yes, No, and Cannot Determine (CD) or Not Applicable (NA) or Not Reported (NR) by the reporters. The reported domains measured confounding, measurement of exposure, selection of participants, post exposure interventions, missing data, measurement of outcome and reporting of results.

**Figure 2:**
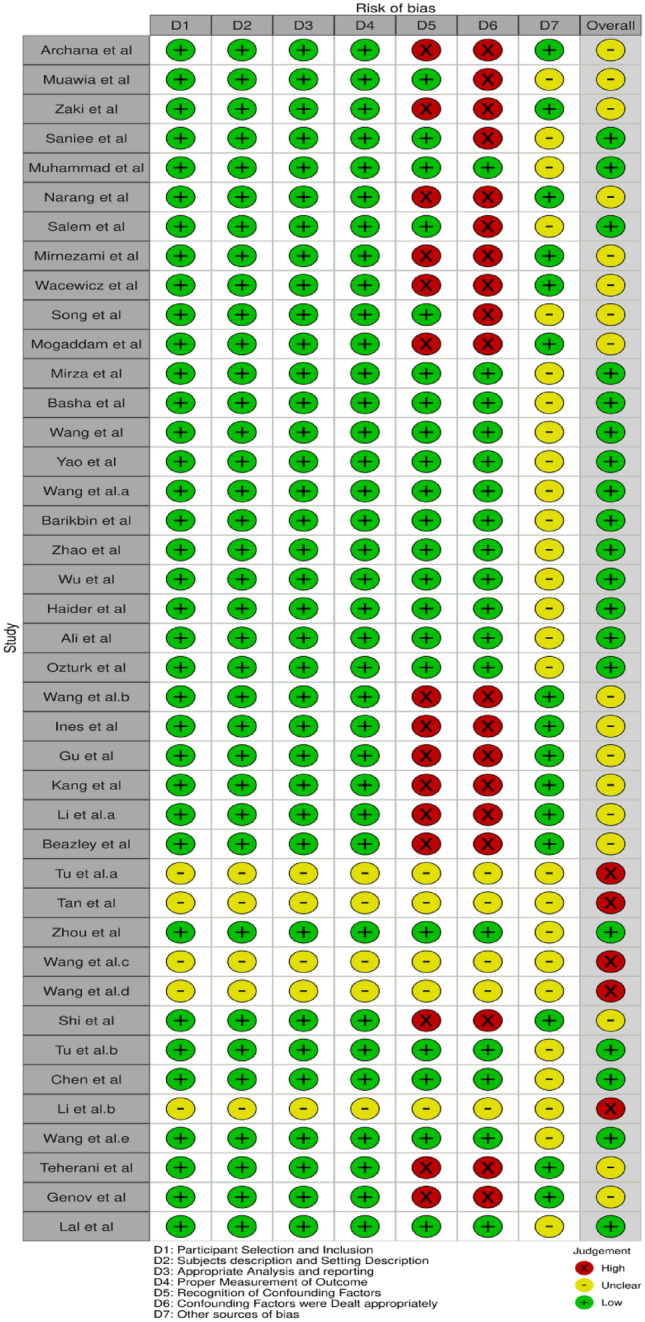
Traffic signal plot for included studies.

The risk of bias was further represented using the RobVis tool for schematically representing the risk of bias from the domains of various studies as a traffic signal plot (Fig. 2).

### Statistical analysis

The presence of any association or correlation between change in serum levels of copper, selenium and Zinc and vitiligo was summarized as means and standard deviations which were further compared by odds ratios (OR) in 95% confidence intervals (CI). Review Manager (RevMan) Version 5.4 (Copenhagen: The Nordic Cochrane Centre, The Cochrane Collaboration, 2014) was used for generation of the weighted forest plot. Heterogeneity was assessed statistically using I^2^ and Cochrane Q statistic.For the assessment of publication bias, Begg’s and Egger’s test was performed using MedCalc Version 20.210 . A *P*-value of ≤ 0.05 or 5% was considered statistically significant. In the absence of a publication bias and statistical heterogeneity (I^2^ < 50%), estimation of a pooled effect by fixed-effect model using the Mantel–Haenszel method was carried out.

## Results

### Study characteristics

This Meta analysis includes studies published between 1970 and 2022.

It was found that twenty-seven studies showed serum zinc levels in vitiligo patients. Geographically, 14 studies were from China, 4 from Egypt, 3 from Iran and Bangladesh and 1 each from India, Poland and Sudan (Table 2).

**Table 2:** Characteristics of studies showing serum zinc levels in patients with vitiligo and healthy individuals included in Meta analysis.

N	Author (References)	Year	Country	Zinc					
				Cases			Controls		
				M*	SD**	T***	M*	SD**	T***
1	Zaki^7^	2020	Egypt	50.93	11.02	50	77.09	12.16	50
2	Saniee^14^	2019	Iran	90.94	20.88	98	84.68	21.08	98
3	Muhammad^13^	2019	Sudan	104.0	20.1	60	93.2	19.5	60
4	Narang^18^	2018	India	24.4	10.8	100	159.4	70.8	60
5	Salem^19^	2018	Egypt	37.16	9.16	50	50.49	11.02	50
6	Mirnezami^9^	2018	Iran	81.3	12.7	63	103	91.8	103
7	Mogaddam^40^	2017	Iran	80.11	17.10	100	96.10	16.16	100
8	Wacewicz^20^	2017	Poland	0.848	0.120	50	0.997	0.292	58
9	Mirza^41^	2016	Bangladesh	74.17	24.70	60	95.50	27.30	50
10	Basha^42^	2014	Egypt	104.0	20.1	60	93.2	19.5	60
11	Wang^31^	2012	China	12.79	2.31	120	13.02	3.53	120
12	Yao^32^	2011	China	0.88	0.26	90	1.07	0.31	50
13	Wang^26^	2011	China	9.9	0.51	28	15.62	2.94	30
14	Wu^43^	2010	China	6.416	1.758	70	7.194	1.412	70
15	Haider^12^	2010	Bangladesh	1.08	0.07	30	0.95	0.35	30
16	Ali^44^	2010	Bangladesh	30.27	11.09	60	32.27	11.08	60
17	Wang^25^	2007	China	0.61	0.05	68	1.31	0.33	20
18	Kang^35^	2002	China	0.9	0.51	30	1.06	2.25	34
19	Beazley^11^	1999	UK	104	20.1	60	93.2	19.5	60
20	Tan^23^	1997	China	17.45	3.56	37	22.85	3.09	750
21	Zhou^27^	1996	China	60.08	31.33	26	97.17	22.54	20
22	Wang^24^	1996	China	74.23	18.99	48	97.4	13.8	141
23	Wang^30^	1993	China	0.9	0.51	30	1.06	2.25	34
24	Shi^45^	1993	China	15	3.81	120	17.21	5.51	150
25	Tu^46^	1991	China	11.5	3.15	22	13.54	2.34	36
26	Chen^47^	1989	China	118.7	30.9	60	151.9	46.9	30
27	Li^29^	1988	China	0.88	0.25	7	1.2	0.45	135

*Mean **Standard Deviation ***Total.

Twenty-three studies showed serum copper levels in vitiligo patients. Geographically, 16 studies were from China, 3 from India, and 1 each from Egypt, Bulgaria, Poland and Sudan (Table 3).

**Table 3:** Characteristics of studies showing serum copper levels in patients with vitiligo and healthy individuals included in Meta analysis.

S. No	Author (References)	Year	Country	Copper					
				Cases			Controls		
				Mean	SD**	T***	Mean	SD**	T***
1	Archana^66^	2021	India	59.8	68.4	60	55.4	37.7	60
2	Muawia^17^	2020	Sudan	21.69	5.17	50	18.05	3.51	50
3	Narang^18^	2018	India	32.5	10.3	100	24.2	6.7	60
4	Salem^19^	2018	Egypt	141.62	32.56	50	128.38	29.03	50
5	Wacewicz^20^	2017	Poland	1.099	0.273	50	1.038	0.336	58
6	Wang^31^	2012	China	13.1	2.56	120	14.78	2.4	120
7	Yao^32^	2011	China	0.69	0.15	90	1.12	0.22	50
8	Wang^26^	2011	China	18.95	0.39	28	19.35	4.32	30
9	Wu^43^	2010	China	1.46	0.471	70	1.536	0.345	70
10	Wang^31^	2007	China	0.81	0.12	68	1.06	0.38	20
11	Gu^28^	2005	China	0.6107	0.0917	30	1.0156	0.4367	36
12	Kang^35^	2002	China	0.88	0.71	30	1.13	0.21	34
13	Li^48^	2001	China	0.807	0.143	96	1.091	0.181	30
14	Tan^23^	1997	China	16.23	1.76	37	22.1	2.19	750
15	Zhou^27^	1996	China	103.74	24.29	26	122.9	39.21	20
16	Wang^24^	1996	China	107.6	24.29	26	122.9	39.21	20
17	Wang^30^	1993	China	0.88	0.17	34	1.13	0.21	30
18	Shi^45^	1993	China	12.21	4.36	120	12.17	4.13	150
19	Tu^46^	1991	China	13.05	2.74	27	14.68	2.33	36
20	Chen^47^	1989	China	88.2	25.47	60	99.66	15.49	30
21	Li^29^	1988	China	0.86	0.13	7	0.95	0.19	135
22	Genov^16^	1972	Bulgaria	129	33	84	99	19	20
23	Lal^49^	1970	India	121.70	29.24	30	126.75	27.07	20

**Standard Deviation ***Total participants.

Twelve studies which showed serum selenium levels in vitiligo patients. Geographically, 6 studies were from China, 1 each study were from Austria, Iran, Poland, Turkey, Tunisia, and UK (Table 4).

**Table 4:** Characteristics of studies showing serum Selenium levels in patients with vitiligo and healthy individuals included in Meta analysis.

S. No	Author (References)	Year	Country	Selenium					
				Cases			Controls		
				M*	SD**	T***	M*	SD**	T***
1	Song^36^	2017	China	0.11	0.02	62	0.16	0.05	62
2	Wacewicz^20^	2017	Poland	51.30	13.99	50	79.42	18.97	58
3	Barikbin^34^	2011	Iran	1.021	0.31	60	0.909	0.07	45
4	Zhao^50^	2011	China	121.9	46.16	36	129.27	23.67	16
5	Yao^32^	2011	China	0.09	0.03	90	0.14	0.07	50
6	Ozturk^38^	2008	Turkey	122.33	30.17	30	120.77	21.8	30
7	Ines^51^	2006	Tunisia	1.52	0.14	18	0.93	0.07	40
8	Kang^35^	2002	China	0.10	0.02	30	0.13	0.04	34
9	Beazley^11^	1999	UK	1.27	0.32	61	0.93	0.20	5932
10	Tu^37^	1998	China	99.41	14.93	29	101.13	12.87	37
11	Wang^30^	1993	China	0.10	0.02	34	0.13	0.14	28
12	Teherani^52^	1986	Austria	0.388	0.044	5	0.304	0.087	8

*Mean **Standard Deviation ***Total participants.

### Overall findings of meta-analysis

#### Levels of serum zinc and its association with vitiligo

**Figure 3:**
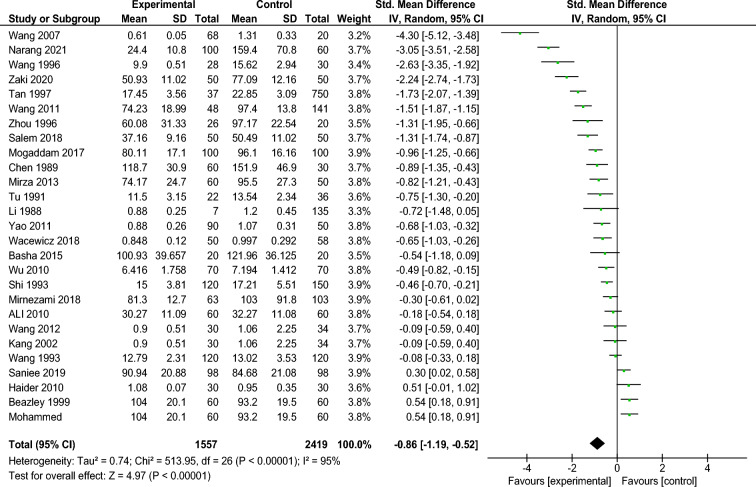
Meta analysis of the serum Zn level between the two groups Vitiligo patients (Experimental) and controls. Compared with the control, there was a significant decrease of Zn level in the vitiligo group (Z = 4.97, *P* < 0.00001; standardized mean difference, − 0.86; 95% confidence interval [CI] − 1.19 to − 0.52). SD, standard deviation.

Out of the 27 investigations reported, the serum Zn levels, eleven studies announced no significant Zn level change between the vitiligo group and the healthy control group. The others introduced critical abatement of Zn level in the vitiligo group. Heterogeneity among the 27 articles was examined first. The results revealed a high statistical heterogeneity among studies (Tau2 = 0.74; Chi2 = 513.95, d.f. = 26 [*P* < 0.00001]; I2 = 95%). Then, a random effect model was utilized for meta-analysis. In the collective examination, there was a critical decline of Zn level in the vitiligo group (Z = 4.97; *P* < 0.0001; SMD = − 0.86; 95% CI − 1.19 to − 0.52; Fig. 3).

#### Levels of serum copper and its association with vitiligo

**Figure 4:**
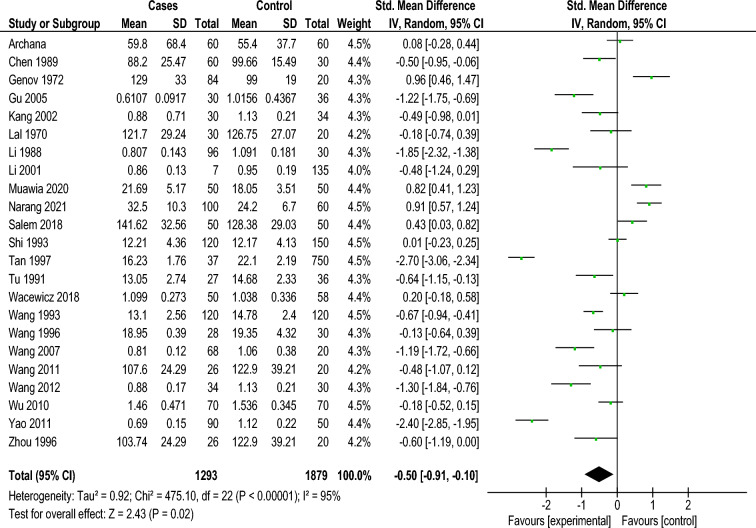
Meta analysis of the serum Cu level between the two groups Vitiligo patients (Experimental) and controls. Compared with the control, there was a significant decrease of Cu level in the vitiligo group (Z = 2.43; *P* < 0.0001; SMD = − 0.50; 95% confidence interval [CI] − 0.91 to − 0.10). SD, standard deviation.

Of the 23 investigation that distinguished the serum Cu level, thirteen investigations reported no statistically significant Cu level change between the vitiligo group and the healthy control group. The others introduced huge abatement of Cu level in the vitiligo group. Heterogeneity among the 23 articles was examined first. The results revealed a high statistical heterogeneity among studies (Tau2 = 0.92; Chi2 = 475.10, d.f. = 22 [*P* < 0.00001]; I2 = 95%). Then, a random effect model was used for the meta-analysis and in the combined examination, there was again a critical decline of Cu levels in the vitiligo group (Z = 2.43; *P* < 0.0001; SMD = -0.50; 95% confidence interval [CI] − 0.91 to − 0.10) (Fig. 4).

#### Levels of serum selenium and its association with vitiligo

**Figure 5:**
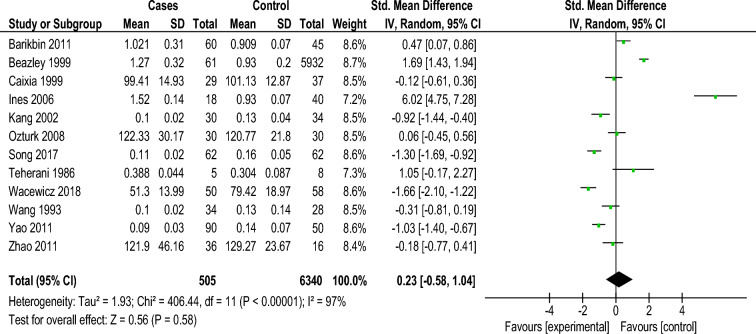
Meta analysis of the serum Se level between the two groups Vitiligo patients (Experimental) and Healthy Individuals (Controls). Compared with the control, there was a significant increase of Se level in the vitiligo group (Z = 0.56; *P* < 0.0001; SMD = 0.23; 95% confidence interval [CI], 0.58 to 1.04).SD, standard deviation.

Among the investigations that reported the serum Se levels, five out of twelve investigations announced no statistically significant Se level change between the vitiligo group and the healthy control group. Two investigations showed slight elevation in sera Se levels, one study reported significant high levels, and four investigations showed decreased levels of selenium in vitiligo patients. Heterogeneity among the 12 articles was examined and the results reveals statistical heterogeneity among studies (Tau2 = 1.93; Chi2 = 406.44, d.f. = 11 [*P* < 0.00001]; I2 = 97%). Then, a random effect model was utilized for meta-analysis and the pooled examination, there was an increment of Se levels in the vitiligo group (Z = 0.56; *P* < 0.0001; SMD = 0.23; 95% confidence interval [CI], 0.58 to 1.04) (Fig. 5).

### Sensitivity analysis and publication bias

The results from 27 studies in serum zinc levels showed Test for Heterogeneity Q = 757.2414, DF = 26, Significance level at *P* < 0.00001; I^2^ = 96.57% and CI at 95% for I^2^ is 95.77 to 97.21.

**Figure 6:**
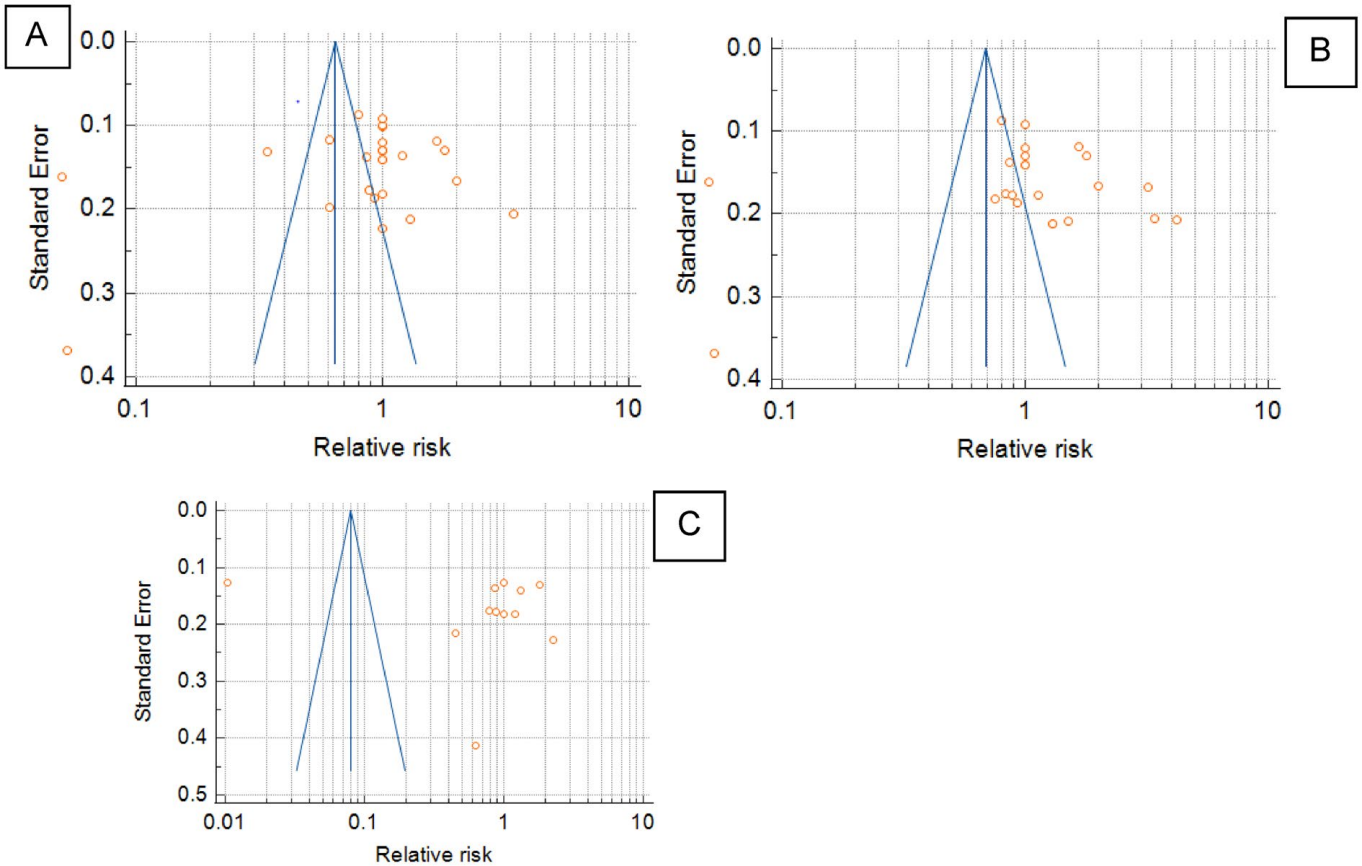
Publication bias for included studies: (**A**) in serum Zinc for Vitiligopatients versus healthy controls; (**B**) in serum copper for Vitiligo patients versus healthy controls; (**C**) in serum selenium for Vitiligo patients versus healthy controls.

For publication bias Egger’s Test showed Intercept at − 2.5534, 95% CI = − 9.5070 to 4.4003 and Significance level *P* = 0.4566; Begg’s Test showed Kendall’s tau = -0.1850 at significance level *P* = 0.1759 [Fig. 6A].

The results from 23 studies on serum copper levels showed Test for Heterogeneity Q = 792.0325, DF = 22, Significance level at *P* < 0.00001; I^2^ = 97.22% and CI at 95% for I^2^ is 96.57 to 97.75.

For publication bias Egger’s Test showed Intercept at − 0.1169, 95% CI = − 8.1912 to 7.9574 and Significance level *P* = 0.9763; Begg’s Test showed Kendall’s tau = − 0.1198 at significance level *P* = 0.4236 [Fig. 6B].

The results from 12 studies on serum selenium levels showed Test for Heterogeneity Q = 3010.2988, DF = 11, Significance level at *P* < 0.00001; I^2^ = 99.63% and CI at 95% for I^2^ is 99.58 to 99.69.

For publication bias Egger’s Test showed Intercept at 8.0349, 95% CI = − 18.6917 to 34.7615 and Significance level *P* = 0.5181; Begg’s Test showed Kendall’s tau = − 0. 1818 at significance level *P* = 0.4106 (Fig. 6C).

## Discussion

Vitiligo is an autoimmune disorder that causes depigmentation of the skin, hair, and mucous membranes. The condition has been described in ancient medical texts, indicating that humans have been aware of vitiligo for thousands of years. Despite this long history, the precise mechanisms underlying the development and progression of vitiligo are still not fully understood, and there exists significant amount ongoing research enabling better comprehension of the condition. Even though the exact cause has not been understood, but individual studies have shown that oxidative stress and trace elements such as zinc, copper, and selenium play a significant role in its pathogenesis. This meta-analysis has multiple exclusive advantages, where by far this is the largest meta-analysis performed on the association of zinc, copper and selenium serum levels and vitiligo. This study does not derail into other antioxidants and provides a specific perspective of metallo-micronutrients and the various possible pathogenetic mechanisms of vitiligo.

These specific micronutrients were chosen due to extensive existing but contradictory evidences present among them, where most studies reflect only on their anti-oxidant or free radical stress pathways of these micronutrients. This study also analyses a subgroup analysis for Asian and Caucasian populations separately, as previous meta-analyses by Chen et al. focuses only on Chinese populations, with high pre-existing risk of skin infections as reported by Lv et al.^8,53^.

### Association between oxidative stress and zinc/copper ratio

Oxidative stress is known to damage cells by generating free radicals that can cause DNA damage, protein oxidation, and lipid peroxidation. A study has reported an association between oxidative stress and the ratio of zinc and copper (Zn/Cu) in vitiligo patients. The study showed that patients with vitiligo had a higher Cu/Zn ratio compared to healthy controls^20^. Zinc and copper are known to be involved in the defence mechanisms against free radicals by comprising Cu/Zn superoxide dismutase, which catalyzes the dismutation of reactive oxygen species to O2 or H2O2. Bagherani et al.^6^ and Yaghoobi et al.^54^ highlighted the association between among vitiligo and Zinc, due to its anti-apoptotic activity, has been suggested to be useful in the control of vitiligo^55,56^.Zinc deficiency can also impact the innate and adaptive immune system by affecting the survival, proliferation, and maturation of monocytes, polymorphonuclear, natural killer T and B-cells. Zinc also possesses antioxidant properties that can counter the toxic effects of free radicals^9^. Another study suggest that higher levels of serum zinc levels denote lower levels of Glutathione S-transferase in females^57,58^. Deficiency of zinc causes the suppression of non-specific cell mediated immunity, so zinc may cause the stimulation of cell mediated immunity against the infective and other probable factors which contributes to the development of vitiligo. α-melanocytes hormone is responsible for the melanogenesis and zinc plays a key role in the synthesis and release of α-melanocytes hormone. Regulation in normal and malignant melanocytes production is by ZAG (zinc-α2-glycoprotein), and zinc precipitates the ZAG at the vitiligo patch site and can be effective in the treatment of vitiligo^59,60^.

### Role of copper and zinc in melanogenesis

Both copper and zinc are integral parts of many metalloenzymes necessary in the process of melanogenesis^20^. Melanin protein, responsible for imparting pigment to the skin, needs the amino acid tyrosine and the enzyme, tyrosinase for its synthesis^61^. The synthesis of melanin is initiated for the conversion of tyrosine to dihydroxyphenylalanine (DOPA), a key regulatory enzyme called tyrosinase. Copper is indispensable for the action of this enzyme, hence playing an essential role in melanogenesis ^7^. Zinc is also necessary for the proper functioning of tyrosinase and melanogenesis, making it crucial for the maintenance of normal skin pigmentation^62^.

### Selenium, glutathione and vitiligo

Selenium is a chemical element that occurs naturally and has been extensively studied in medicine and biology due to its nutritional effects. The liver produces a protein called selenoprotein P (SELENOP), which is the primary component of circulating selenium. SELENOP provides essential tissues, such as endocrine glands and cells expressing insulin receptors, with the necessary amount of selenium. In tissues and hair, selenium is found in various forms, including selenocysteine, selenomethionine, selenoproteins, and other selenium-containing proteins and enzymes. Selenium plays a crucial role in the synthesis of selenoproteins, which have a wide range of pleiotropic effects, including antioxidant and anti-inflammatory effects. Several selenoproteins possess antioxidant properties that can help protect the human body against diseases caused by free radical damage, including malignant, infectious, and cardiovascular diseases^63^. The association between serum levels of copper, zinc, and vitiligo is still unclear due to conflicting results reported in various studies. Glutathione peroxidase is a selenium-containing enzyme that helps protect cells against oxidative damage. Together, selenium and glutathione peroxidase play a critical role in maintaining cellular health and preventing a range of diseases associated with oxidative stress. Previous meta-analyses have investigated the importance of selenium levels and glutathione peroxidase (GPX) levels in vitiligo^11,34,38^.

Our pooled analysis showed that the overall selenium level was similar between vitiligo patients and healthy controls, but a subgroup analysis unveiled that selenium levels were slightly higher in vitiligo patients than in healthy controls in the Asian population. This suggests that high selenium levels may contribute to the pathogenesis of vitiligo in the Asian population. Race could also be the reason for high selenium levels in the Asian population, as it is a structural component of the GPX enzyme. This falls in similar conclusions as observed by the systematic review by Huo et al. indicated that vitiligo patients observed a higher serum concentration of vitiligo in line with the existing biological evidence in mice models^64,65^. In contrast,systematic review by Lv et al.^53^ shows low serum selenium levels to be associated with vitiligo. This lack of congruence on selenium levels and associated risks needs to be further evaluated by studies for viable treatment options. This study bears some limitations; the study involves a large pooling of data on a controversial yet significant medical pathology. Vitiligo treatment modalities have been largely experimental as the evidence has not been concordant so far. This study draws the associations of serum micronutrient levels and vitiligo among patients and controls over large populations along with sub-group analysis specific to Asian population to enable treatment personalization and further incentive to perform high quality research. The study is limited by the limited number of databases it had access to, and may have missed out on studies that may have tilted the final outcome. This study also acknowledges the limited statistical conclusivity that exists due to the discordant nature of studies’ results.

## Conclusion

The pathogenesis of vitiligo is complex and involves oxidative stress, trace elements such as zinc, copper, and selenium, and melanogenesis. There exists a significant decline in serum micronutrient levels in patients of vitiligo as compared to individuals who were taken as healthy controls. A more significant association exists as per sub-group analysis among the Asian population with serum selenium levels (reduction) as compared to overall reductions. Although the association between these trace elements and vitiligo is not yet fully understood, this article highlights the current understanding of their role in the disease. Further research is needed to elucidate the mechanisms underlying the association between these trace elements and vitiligo and to develop effective therapies for this debilitating condition.

## Data Availability

The datasets used and/or analyzed during the current study available from the corresponding author on reasonable request.
